# Gels/Hydrogels in Different Devices/Instruments—A Review

**DOI:** 10.3390/gels10090548

**Published:** 2024-08-23

**Authors:** Md Murshed Bhuyan, Jae-Ho Jeong

**Affiliations:** Research Center for Green Energy Systems, Department of Mechanical, Smart, and Industrial Engineering (Mechanical Engineering Major), Gachon University, 1342 Seongnam-daero, Sujeong-gu, Seongnam-si 13120, Gyeonggi-do, Republic of Korea

**Keywords:** hydrogel, device, sensor, actuator, touch panel, solar cell, battery, soft robotics

## Abstract

Owing to their physical and chemical properties and stimuli-responsive nature, gels and hydrogels play vital roles in diverse application fields. The three-dimensional polymeric network structure of hydrogels is considered an alternative to many materials, such as conductors, ordinary films, constituent components of machines and robots, etc. The most recent applications of gels are in different devices like sensors, actuators, flexible screens, touch panels, flexible storage, solar cells, batteries, and electronic skin. This review article addresses the devices where gels are used, the progress of research, the working mechanisms of hydrogels in those devices, and future prospects. Preparation methods are also important for obtaining a suitable hydrogel. This review discusses different methods of hydrogel preparation from the respective raw materials. Moreover, the mechanism by which gels act as a part of electronic devices is described.

## 1. Introduction

**Figure 1:**
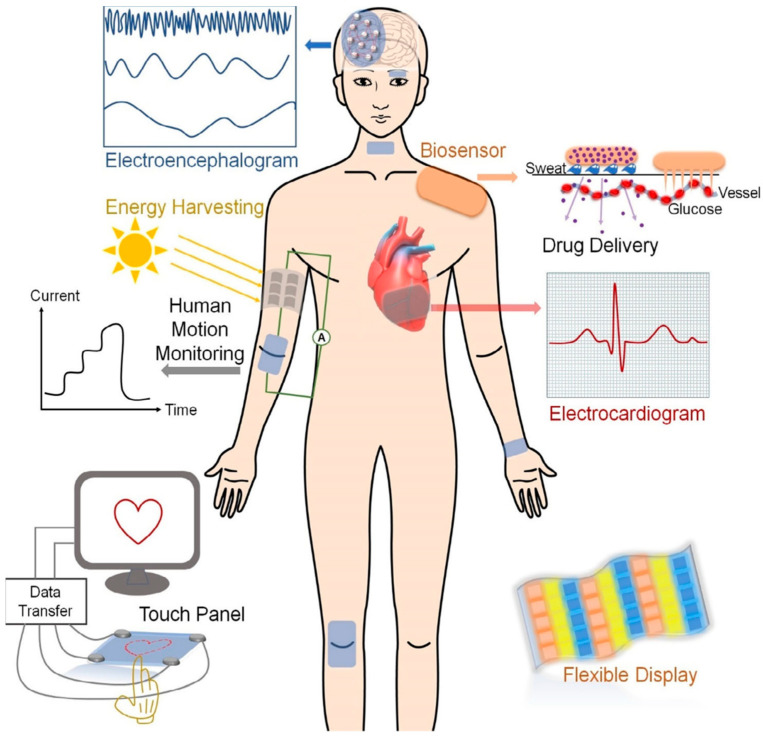
Conductive hydrogels used in different devices (reused with permission [35]).

The three-dimensional soft, solid, or solid-like network prepared by chemical or physical cross-linking between polymers through the copolymerization method is referred to as gel. When the dilute solution of polyvinyl chloride phthalate is cooled, it turns into gel. Gels may absorb or swell in aqueous (water) or non-aqueous (organic) solvents. Styrene–divinylbenzene is a covalently cross-linked gel swell in an organic solvent. Silica gel is an inorganic gel that absorbs and swells in water. S.P. Papkor first reported gels in 1974, and later, T. Tanaka did a survey on polymer gels [1,2]. When a three-dimensional polymeric network (gel) can retain a large amount of water without dissolving, it is referred to as a hydrogel, and if the hydrogel can absorb water 1000 times its dried weight, it is classified as a superabsorbent hydrogel [3,4]. When the gels contain high porosity with extended mechanical strength and a higher surface area for greater adsorption capacity, they are termed aerogels. Aerogels may possess metal oxide, graphene/graphene oxide, and carbon nanotubes to make themselves sustainable [5]. Due to having different functional groups, like -COOH, -OH, -NH_2_, -NHR, -SO_3_, -PO_3_, etc., and cross-linking or grafting between entangled networks, hydrogels show swelling and multi-functional activities. On the basis of structural moieties, preparation methods, and functional groups present in the network, they show stimuli-responsivity towards pH, temperature, pressure, electric and magnetic fields, light, and ionic strength (salt effect), which facilitates hydrogels being used as sensors in devices [6]. The mechanical strength, electrical conductivity, and biocompatibility of hydrogels make them suitable for use in actuators and biomedical engineering. Their multi-network structure and self-healing properties allow hydrogels to become usable in flexible devices. In addition, hydrogels are widely used in water purification [7], metal and dye adsorption, drug delivery, water and fertilizer supply in agriculture fields, and wound dressing [8]. Both natural (starch, pectin, cellulose, chitosan, and dextrin) and synthetic polymers (polyvinyl alcohol, polyvinyl chloride, etc.) are widely used for cross-linking or grafting with different monomers, subsequently producing hydrogels of diverse physical and chemical natures. By varying the conditions, such as the composition of raw materials, radiation dose (for radiation-induced hydrogels), and the network’s layer, different types of monomers and metal components can be tuned to fit arbitrary parts of devices. The tuning is performed depending on the purposes of the application [9]. For example, Shuwang et al. reported poly(vinyl alcohol) hydrogels whose mechanical properties are tuned for wearable electronics [10]. For bioelectronic adhesion, water-resistant hydrogels were prepared by tuning the hydrophobic alkyl chain [11]. Ionic gels possess a conductive organic polymer backbone, an ionic liquid (sometimes metallic compounds), and free-moving ions (electrons) throughout the networks, which enable the gels to be used as conductors in different devices [12]. Metal conductors’ limitations required researchers look for new types of conductors with flexibility, self-healing, and soft mechanical and biocompatible properties, leading to the invention of bioelectronics and soft devices [13]. The devices that the gels are used in include sensors [14], actuators [15], soft robotics [16], flexible energy storage [17], solar cells [18], touch panels [19], and electric skin [20]. Despite the progress of gels as a part of devices, they face conductance limitations and efficiency challenges. More emphasis has to be imposed to mitigate and improve the conductive gels for use in different targeting devices. Polymers are usually used to make various gel components of devices. These polymers include poly(3,4-ethylenedioxythiophene)–polystyrene sulfonate (PEDOT:PSS) [21,22], tetrathiafulvalene (TTE) [23], polyaniline (PANI) [24,25], polypyrrole (PPy) [26], 7,7,8,8-tetracyanoquinodimethane (TCNQ) [27], poly(3,4-ethylenedioxythiophene), polythiophene (PTh) [28], phenylene vinylene [29], polycarbazole [30], chitosan [31], cellulose [32], starch [33], pectin [34], and some other polymers. To improve the conductivity and self-healing properties of hydrogels, different metal oxides, graphene oxide, carbon nanotubes, and salts are incorporated with conductive organic compounds during the hydrogel preparation, resulting in the upgraded products. Those compounds include TiO_2_, CaO, MgO, Fe_2_O_3_, ZnO, CaCl_2_, HAuCl_4_, and NaCl [35,36,37]. Qiongyao et al. [35] explained the use of conductive hydrogels in different devices required for the human body and other fields, as shown in Figure 1. The most important part of conductive gels is the pie-conjugation structure of the conductive polymer, which favors electricity conduction. The combination of a conjugation system and metallic supports improves the gel quality of devices [38]. To improve gels’ performance in different devices, researchers are trying to fabricate and develop the best raw materials and optimize the radiation dose. Moreover, new materials could be developed to impart breakthrough changes in this sector. However, this review explains the involvement of various gels in different devices and instruments necessary for our daily practical lives. 

## 2. Gels/Hydrogels

### 2.1. Properties of Gels/Hydrogels

**Figure 2:**
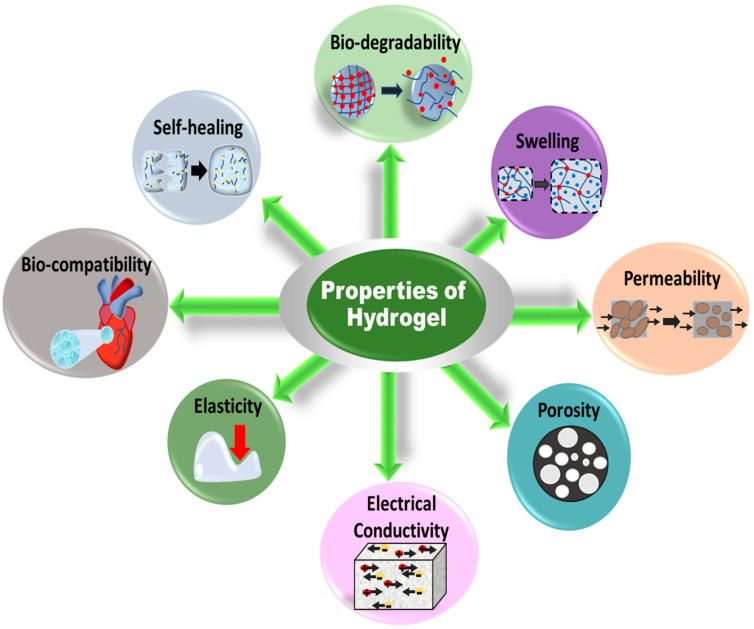
Properties of hydrogels for application to different devices.

**Figure 3:**
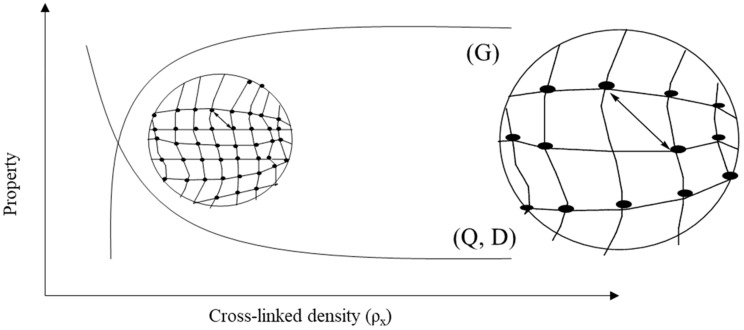
Property versus cross-linked density graph for correlating shear modulus, (G) swelling ratio (Q), and molecular diffusivity (D).

The properties of gels depend on the nature of the network, types of cross-linking and grafting, and functional groups. Jun et al. briefly mentioned most of the properties of hydrogels possessing low–high viscosity, hydrophilicity–swelling, responsivity to pH, pressure, temperature, light and sound, electric and magnetic fields, conductivity, self-healing, biodegradability, biocompatibility, porousness, and flexibility [39], as shown in Figure 2. Tunable hydrogels are receiving attention for use in devices. The mechanical properties and water-holding capacity can be controlled by altering the cross-linking density and functional groups on the hydrogel network. Tuning in the cross-linking density is a function of changes in stiffness, molecular diffusivity (*D*), mess size (*ξ*), gel mechanics (shear modulus, *G*), and swelling ratio (*Q*) of the polymeric networks. Figure 3 presents the property versus cross-linking density (*ρ_x_*), where the shear modulus increases and molecular diffusivity and swelling ratio decrease with increasing the cross-linking density [40]. The correlation among the quantities can be expressed as the following equation [41]:

(1) $$G=RT\rho_{x}Q^{-\frac{1}{3}}$$

where *R =* the universal gas constant and *T =* the temperature. Therefore, *G* is measurable from the equation above. The determination of diffusivity is important for evaluating the swelling character. According to Peppas’s explanation, the molecular diffusivity and network structure of the hydrogel can be related by

(2) $$D=D_{o}\left(1-\frac{r_{s}}{\xi }\right)e^{-Y(\frac{Q}{Q-1})}$$

Here, *D_o_* = the molecular diffusivity in a pure solvent, *r_s_* = the hydrodynamic radius of the diffusing particle, and *Y* = the ratio of the critical volume and the average free volume per molecule of solvent; usually, it is considered unity.

### 2.2. Fabrication and Structure of Gels/Hydrogels

**Figure 4:**
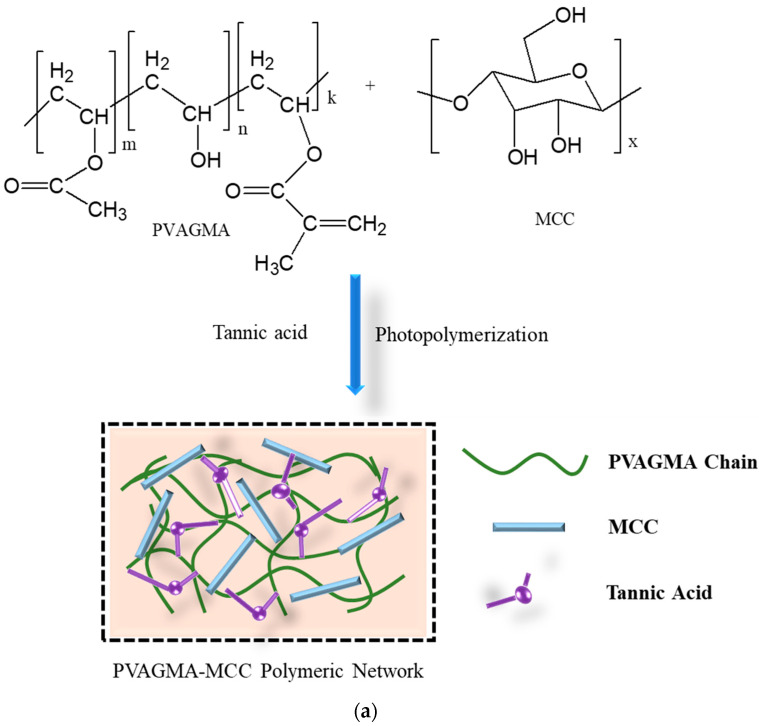

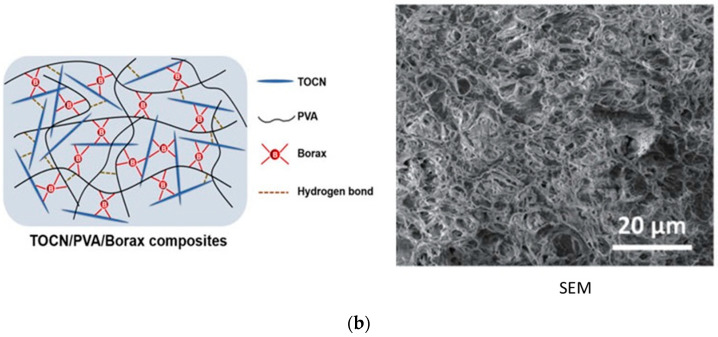
Fabrication and structure of (**a**) PVAGMA-MCC hydrogel cross-linked by tannic acid and (**b**) borax cross-linked TOCN/PVA composite gel and its SEM photograph [43].

Hydrogels possess a three-dimensional polymeric network formed by chemical or physical cross-linking [42]. The structure of gel can be entangling, porous, or, in some cases, crystalline [43,44]. Hydrogels are formed by the cross-linking or grafting of polymers and monomers through the cross-linking agent. In the solution method of polymerization, a cross-linking agent and reaction initiator are added, which remain in the structure after synthesis, resulting in impurity of the products. The radiation method does not require initiators and cross-linking agents because irradiation initiates polymerization by producing free radical points on the backbone of the polymer and vinylic parts of the monomer, followed by propagation and termination [45]. Minjie Pei et al. [46] reported a microcrystalline cellulose-fabricated double cross-linked poly(vinyl alcohol)–glycidyl methacrylate (PVAGMA-MCC) hydrogel where tannic acid was used as a cross-linking agent, as shown in Figure 4a. Figure 4b exhibits the structure and surface morphology (SEM) of a TEMPO-oxidized cellulose nanofiber-fabricated polyvinyl alcohol (TOCN-PVA) composite gel cross-linked by the inorganic cross-linker borax [47]. Both chemical and physical cross-linking methods contribute to the diverse properties and applications of hydrogels.

### 2.3. Application of Gels/Hydrogels

**Figure 5:**
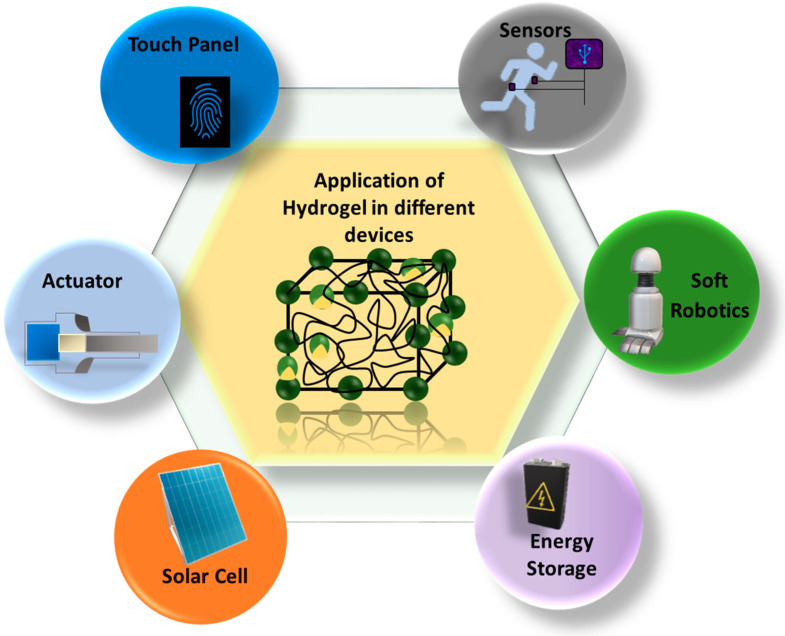
Application of hydrogels in different sectors.

Many modern electronics require soft, flexible, biodegradable, and biocompatible compounds for their assembly, leading to smart devices. In this sector, different types of gels are becoming valuable candidates. There are diverse applications of gels/hydrogels, including adsorption [48], agriculture, medical science, electrical and electronics engineering, coating, optics, and water harvesters [49]. Figure 5 shows the application of gels in various devices, such as sensors, actuators, flexible batteries, solar cells, touch panels, and robots. A lot of work has gone into improving these hydrogels’ mechanical characteristics in recent years so that they may be employed as anatomical and physiological structural supports. Hydrogels can mimic some properties of living tissue, which makes them applicable for tissue engineering and biosensing [40]. It has been proven that gels and hydrogels with stimuli-responsivity are promising 3D polymeric networks for sensor design and implementation [50]. The ordinary actuators in robots or other instruments are brittle and incompatible with biological environments. On the other hand, hydrogels can retain 90% of water, in addition to their other favorable properties, such as an undistorted structure, mechanical strength, and biocompatibility. That is why hydrogels are considered an alternative to traditional actuators [51]. Electrically conductive hydrogels are responsible for the subsequent bending, twisting, and stretching qualities that make up the latest technology in flexible energy storage [52]. An inorganic–organic combination of raw materials is blended to synthesize hydrogels for an efficient approach in touch panels and solar cells [53,54]. A revolutionary change has been brought to soft robotics by applying soft double- or triple-network hydrogels [55].

### 2.4. Choice Preparation Method of Gel/Hydrogel for Devices

**Figure 6:**
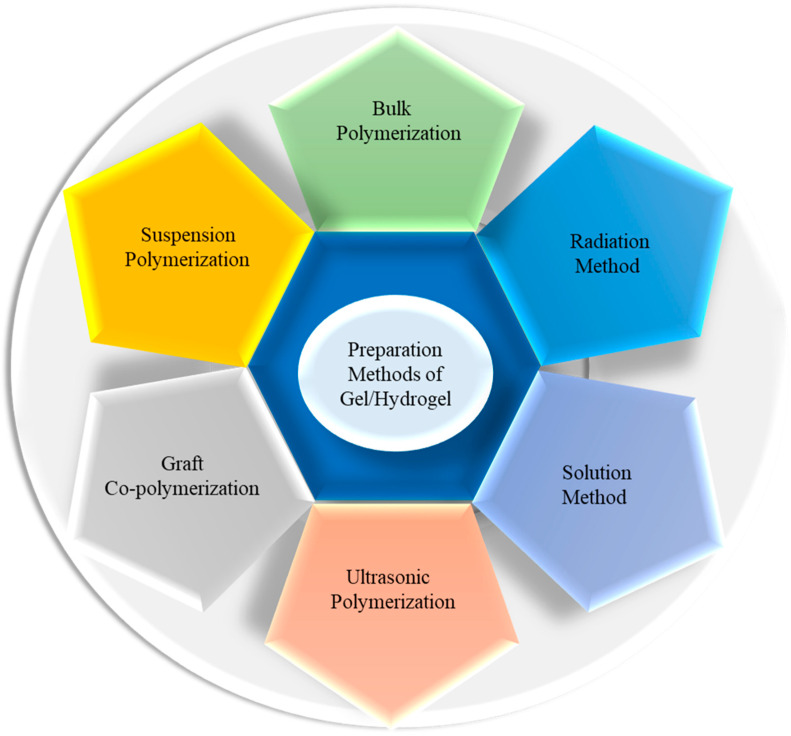
Different preparation methods of gel/hydrogel.

Since specific devices have definite requirements for choosing hydrogels to be used as a part, it is important to select the appropriate raw materials and preparation methods. The preparation method and components of hydrogels determine the characteristics; for instance, the presence of sulfonic groups responsible for the selective adsorption of trivalent metal ions from multi-element solutions on pectin–acrylamide-(2-acrylamido-2-methyl-1-propanesulfonic acid) hydrogels prepared by applying the gamma radiation technique [56]. Here, the grafting of monomers on pectin chains was exerted without using cross-linking agents or initiators, leading to the formation of pure hydrogels of monomers and polymers. For drug delivery, gels should be made of specific biocompatible, biodegradable materials using a reliable preparation method. The different methods of hydrogel preparation are shown in Figure 6. Mutually miscible monomers and initiators are used to prepare hydrogels through bulk polymerization, which produces glassy, hard, and transparent products, as well as heat energy. Yawen et al. monitored sweat glucose using wearable MXene (Ti_3_C_2_Tx) functionalized PEDOT:PSS hydrogels [57]. The solution method uses ionic or neutral monomers and cross-linking agents with multi-functionality. The reaction is started by providing thermal energy and UV radiation, where the solvents (water, ethanol, benzyl chloride, etc.) act as heat sinks. Huihui et al. prepared a double network hydrogel from polyacrylamide, zinc (ii), lithium (i), and physically cross-linked *κ*-carrageenan for flexible electronic devices [58]. Suspension polymerization gives powder or amorphous composite gels or beads prepared by dispersing monomers and initiators in hydrocarbons [59]. To prepare hydrogels possessing better mechanical properties and multi-functionality, smaller monomers are grafted on the backbone chain of polymers (natural and synthetic) [60]. A few aqueous viscous solutions of monomers (example: 2-hydroxyethyl methacrylate) and polymers (example: dextran methacrylate) undergo hydrogel formation under ultrasonic sound [61]. Debbi et al. reported an ultrasound-mediated polyvinyl alcohol–methacrylate hydrogel that was exposed to 1 MHz ultrasound without adding precursor linkers or photo-initiators, and applied it to 3D printing and cell and drug delivery [62]. The most promising and mostly usable methods for preparing pure hydrogels are radiation techniques, which involve ultraviolet, microwave, gamma, and electron beam irradiation. Two rays are highly energetic and ionizing and can penetrate the blend solution to initiate polymerization through the formation of free radicals in the respective raw materials [63]. Therefore, based on the specific requirement, one can choose a suitable method for the synthesis of an appropriate hydrogel.

## 3. Gels in Devices

Pioneering applications of hydrogels in devices/instruments use definite stimuli-responsiveness as their working principle. In the last decade, many research articles were reported or published in different journals, and these are reviewed in this section.

### 3.1. Gels in a Sensor

**Figure 7:**
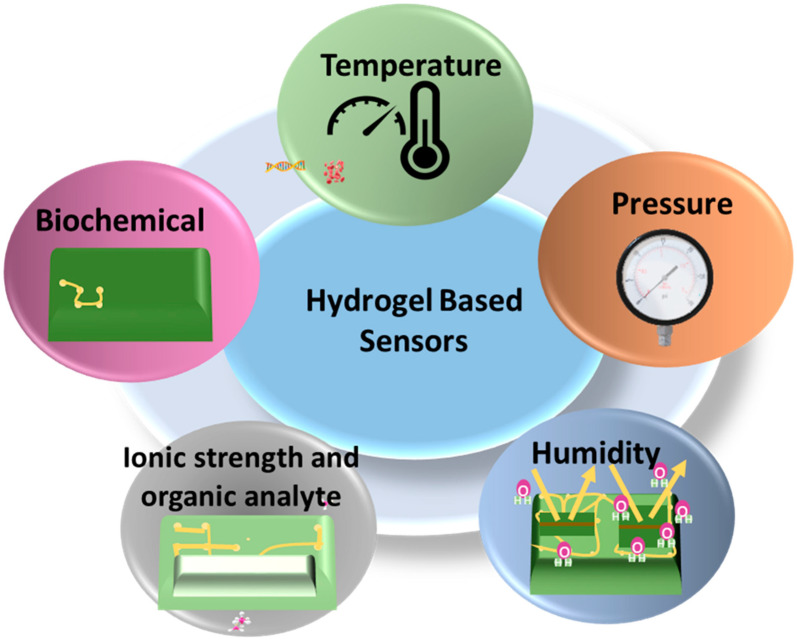
Different hydrogel-based sensors.

A sensor is a type of device that is able to detect and respond to different inputs from the physical environment. The sensor converts energy from one form to another to produce a signal that represents information about the signal [64]. By varying the range and intensity of the stimuli-responsiveness of hydrogels, sensors are prepared for use in biomedical engineering, drug delivery, and other instruments. The sensors most commonly used are pressure and strain sensors [35]. By varying the range and intensity of the stimuli-responsiveness of hydrogels, sensors are prepared for use in biomedical engineering, drug delivery, and other instruments. The sensors most commonly used are pressure and strain sensors [16,65,66]. Recently, several materials have been developed and designed for the purpose of producing flexible sensors, like polyimides [67], conductive polymers [68,69], graphene [70], polyurethane [71], ionic liquids [72], liquid metals [73], etc. However, these sensors often suffer from unsatisfactory sensitivity, insufficient detection range, low stretch ability, and inferior biocompatibility. Since sensors have been used on skin and tissue for a long time, the sensors have to be extremely flexible, biocompatible, nontoxic, and soft so that they can be used safely in the human body [74]. Unlike traditional sensors, hydrogel-based sensors rely on the characteristics of hydrogel, such as high water content, stimulation responsiveness, and high permeability [49]. Figure 7 includes the hydrogel-based wearable sensors that are becoming increasingly significant for biomedical and physiological applications.

The application of conductive hydrogels with excellent self-healing ability in wearable sensors has attracted massive attention in recent years. Hui et al. developed a polyvinyl alcohol–carrageenan self-responsive hydrogel for NO_2_ and NH_3_ sensing. The rapid and reversible mechanism is shown through molecular crystallization, electrostatic interaction, and hydrogen bonding [75]. Zhang et al. designed and developed a supramolecular sodium alginate nano-fibrillar double network hydrogel exhibiting high self-healing, strain sensitivity, and transparency [76]. Liu et al. reported a wearable strain sensor based on a conductive, elastic, self-healing, and highly strain-sensitive CNCs-Fe^3+^-based hydrogel with a soft and hard hierarchical network structure [77]. The hydrogel shows stable electrochemical behavior and self-healing capability within 5 min. A novel conductive polymer hydrogel was reported by Chen et al. and fabricated by cross-linking the 2-ureido-4[1*H*]-pyrimidinone (UPy) group with the polyaniline/poly(4-styrenesulfonate) (PANI/PSS) network [78]. The hydrogel-based strain sensor exhibits external strain and rapid self-healing within 30 s upon damage. Hydrogels’ anti-freezing properties can help to maintain their stability at low temperatures and improve their practical application. Liu et al. were also able to create highly strong, anti-freezing conductive hydrogels for wearable strain sensors using a binary solvent solution of ethylene glycol and H_2_O in order to attain the freezing property [79]. The hydrogel can operate at a temperature of −20 °C. Wang et al. demonstrated a conductive hydrogel-based strain sensor with outstanding anti-freezing properties using poly(3,4-ethylenedioxythiophene):sulfonated lignin as the conducting material and a water/glycerol binary solvent as the dispersion medium [80]. The resulting conductive hydrogel sensors can maintain good mechanical and conductivity properties at −15 °C. Lu et al. designed a mussel-inspired conductive hydrogel with a glycerol–water mixture and a polydopamine-decorated carbon nanotube [78]. The formed hydrogel contains high mechanical properties and flexibility and can recover from deformation at −20 °C to 60 °C. 

**Figure 8:**
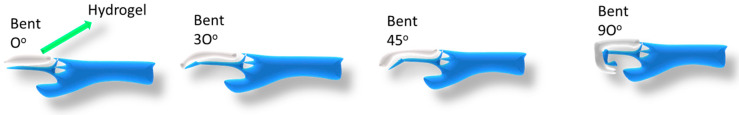
Polyacrylamide (PAAM)/chitosan (CS) hydrogel showing angle of bend for strain sensor.

To fulfill the demand for a stretchable strain or pressure sensor, Shan et al. designed and developed a multifunctional conductive hydrogel composed of a polyacrylamide (PAAM)/chitosan (CS) hybrid network [65]. The PAAM network was cross-linked by hydrophobic association, and the chitosan (CS) network was cross-linked by carboxyl-functionalized multi-walled carbon nanotubes (c-MWCNTs). These two networks are further interconnected by physical entanglement and hydrogen bond interactions. The dynamic cross-linking network provided the hybrid hydrogel with excellent mechanical properties. Figure 8 shows the joint motion of the hydrogel attached to the finger and the resistance during the bending of the finger. The hydrogel resistance progressively rose when the finger was bent step by step (0°, 30°, 45°, 90°). Here, the hydrogel resistance was steady and constant at a certain angle when the finger remained at a consistent value. The hydrogel resistance did not change while the finger was kept in a static position. This shows the precise bending angle measurement through resistance. Furthermore, the hydrogel shows the ability to track small movements, such as speech, breathing, and heartbeat. Table 1 lists a few recent hydrogels used in different sensors whose performance is promising, but the efficiency has to be improved.

### 3.2. Hydrogels in Actuators

**Figure 9:**
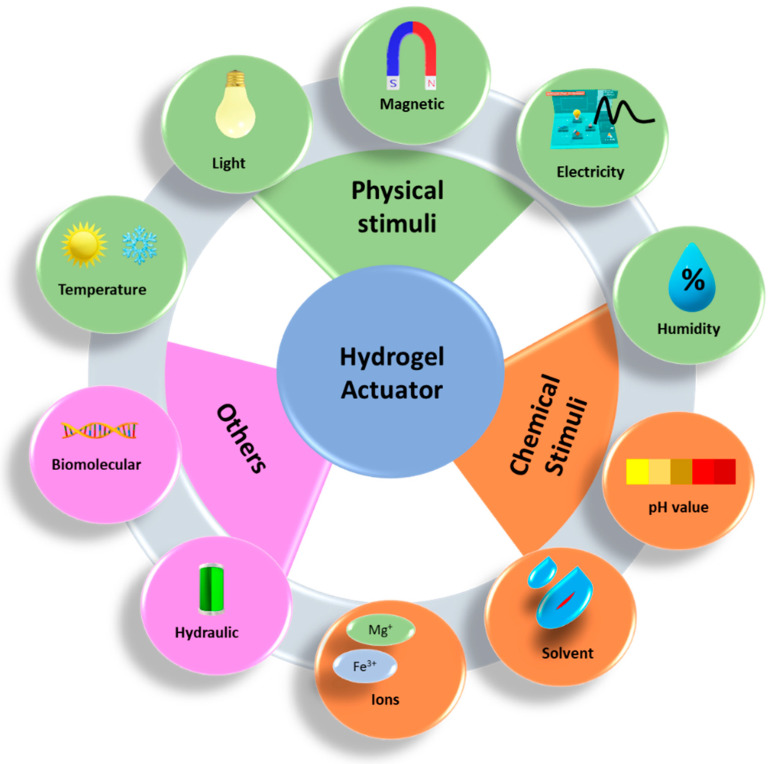
Different stimuli-responsive hydrogel actuators.

An actuator is a type of technology that transforms input energy into usable mechanical energy. With the rapid development of microtechnology, the necessity for the development of devices that can perform mechanical work on a micro- and macroscale has increased [92]. There have been several studies performed on the design of actuators using piezoelectric effects [93,94], fluid flow [95], magnetic fields [96], and electric fields [97]. Among them, stimuli-responsive hydrogels received much attention. Because of its high-water content, biocompatibility, and biometric properties, hydrogel is more advantageous than other materials. Hydrogel material can provide the properties of motion and actuation because of its capacity to expand and shrink in response to the amount of water present or absent. This enclosed water reacts and is sensitive to a variety of outside stimuli, including strain, heat, electricity, and magnetism [98], as shown in Figure 9. For instance, the thermal stimuli response system is extensively used and investigated for artificial intelligence systems, which has demonstrated an important role in the creation of hydrogel actuators.

**Figure 10:**
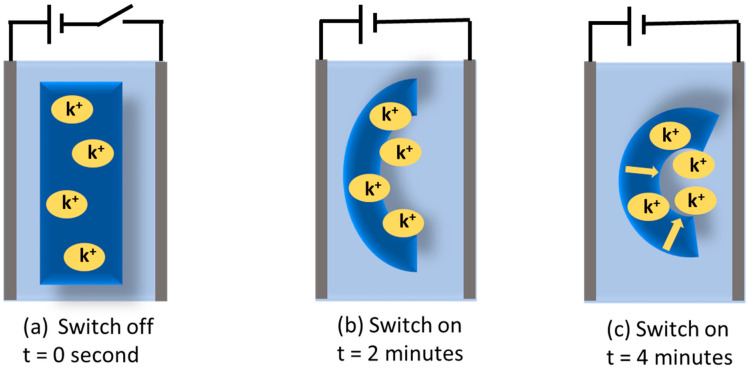
Schematic illustration of PSPA-based hydrogel’s electroactuation on applied electric field. (**a**) At zero time, (**b**) bending characteristics of PASA-based hydrogel at 2 min, and (**c**) at 4 min.

Zhao et al. created a bilayer hydrogel actuator using a PAAM/PAAC layer with the upper critical solution temperature (UCST) effect and a PAAM layer without responsive function [99]. Here, when the temperature drops below UCST, the hydrogel actuator deforms spontaneously. Furthermore, the PAAM layer remains unchanged by temperature. To create smart hydrogels with a lower critical solution temperature (LCST) and UCST-sensitive characteristics, Sun et al. created a temperature-driven hydrogel using poly(N-acrylamide) (PNAGA) layers and PNIPAM layers [100]. Here, at low and high temperatures, the two polymer layers in the hydrogel showed different thermal responses in terms of their expansion and contraction characteristics. This hydrogel actuator could respond quickly to temperature changes and quickly recover from them. Chan et al., inspired by the mimosa plant, prepared a bilayer hydrogel based on the Poly(Acrylic acid-*co*-Acrylamide) layer and the Poly(NIPAM) layer [101]. Here, internal moisture transfer enables the hydrogel actuator to realize the actuation execution in an open-air environment. The light stimulation method is the most-used method for hydrogel actuators because of its long distance, fast, non-contact, and sensitive characteristics. Natural light is one of the most readily available sources and has a wide range of applications for actuators. A lot of research has been conducted on smart hydrogels that are precisely activated by light. Xia et al. produced a PNIPAM-AuNPS/PAAM thin film hydrogel actuator doped with visible light-responsive photothermal gold nanoparticles [102]. The photothermal nanoparticles inside the hydrogel actuator produce heat when simulated by external light through their own photothermal effect. Furthermore, the hydrogel’s deformation is reversible. The reversible actuation performance of natural light shows a critical value in the remote and precise control of the process. Chen et al. created a light-responsive hydrogel actuator using polyurethane (PU) and carbon black (CB) as printing doping materials [103]. Inspired by sunflowers, they made composite artificial multilayer sunflowers. The artificial sunflower has a precise driving procedure like a natural sunflower. Here, the petals of the sunflower are opened at 30 °C. Additionally, the deformation of the hydrogel is reversible. Kang et al. constructed a DC electroactive hydrogel (EAH) actuator based on poly(3-sulfopropyl acrylate potassium salt) (PSPA) by 3D printing, where they used multileg long-chain (MLLC) cross-linker glycidyl methacrylated hyaluronic acid (GMHA). Sulfonate groups are negatively charged and fixed to the actuator hydrogel, but potassium ions are positively charged and move freely when an electric field is applied, leading to electroactivity and actuation. This type of actuator typically has the feature of being able to adjust its volume and structure. As illustrated in Figure 10, when the switch is on and electricity flows towards the PSPA-based hydrogel, potassium (K^+^) ions move in the direction of the cathode to respond to the electric field. Moreover, the remaining anionic sulfonate group is attached to the anode side. As shown in Figure 10, the hydrogel is positioned in the middle of the two electrodes in the KCl solution. Electric osmotic pressure is created at the interface between the sides of the hydrogel when the external circuit is closed because of the electric field’s effect on the ion concentration at that location. The hydrogel’s cations move to the electrolyte solution in order to make the hydrogel electrically neutral. The hydrogel actuator reacts to the electrical field simultaneously by bending towards the negative pole. At 7.5 V, the induced ion waves in the electrodes move at a speed of 1 mm/min, which takes 10 min for actuation. However, GMHA-PASA EAHS takes 4 min at 7.5 volts for actuation, which is substantially faster. The electroactivity increases with increasing the concentration of KCl up to 0.05 M, then starts decreasing. Therefore, the optimum concentration of KCl for electroactuation of the GMHA-PSPA EAHs was found to be 0.05 M. These findings show the adaptability and controllability of the DC hydrogel actuators. Because of its shape-changing ability, the hydrogel actuator can be used in biosensors and artificial muscles [104]. Table 2 refers to some recent hydrogels used as actuators, where the best banding angle is 400° with other properties.

### 3.3. Hydrogels in Touch Panels

In the modern living era, machine and human interactions with touch panels play a significant role due to the daily interaction between various devices like smartphones, laptops, tablets, game consoles, ticketing machines, and various electronic devices [114], as well as their advantages like easy use, portability, and intuitive characteristics [115]. The significant aspiration of touch panels is to obtain factors like biocompatibility, stretchability, softness, high resolution, low parasitic capacitance, fast response, and rapid functionality. For this reason, the stiff and brittle attributes of indium tin oxide (ITO), which is used as a transplant-conducting film in the conventional touch panel, make it constrained. To overcome these limitations, some other options are available, like metal oxides, metal composites [116], carbon nano-materials [117,118], conducting polymers [119], copper nanowires [120], and silver nanowires [121]. Further research is required to prove the biocompatibility of these substitute materials [122].

In the segment of artificial intelligence, wearable devices, and soft robotics, ultra-stretchable and sensitive soft touch panels have significance and a wide range of functions. Nevertheless, conventional human–computer interfaces deteriorate due to displeasurable and asynchronous signals [53]. However, common ionic hydrogels made of chemically cross-linked acrylamide (PAAm) [123] undergo poor surface bonding and lack a self-healing capacity [124]. Zhou et al. made an ionic touch panel from PAAm hydrogel containing LiCl, but the performance was below 500%; the tensile strength, self-healing attributes, and feeble surface bonding were absent [125]. A self-healing semi-conductive touch panel was made by exploiting a novel TiO_2_ nanocomposite hydrogel as the panel material by Guo et al. [115]. The panel exhibited low tensile properties of about 1100%. A capacitive hydrogel-based ionic skin sensor was introduced by Lei et al. that is recyclable, freely self-healable, and designed for curves and dynamic surfaces that are mechanically compliant, although the tensile property was only about 1000%. By using ionic hydrogels made of sodium polyacrylate (SA), poly(vinyl alcohol) (PVA), and sodium tetraborate decahydrate (borax), where borate salt and Na^+^ cation on the polymer chains were used as the charge carriers, Kewon et al. [126] were able to create a stretchable and self-healing touch sensor. Gao et al. expanded a self-feeling ionic touch panel using a polyzwitterion–clay hydrogel, despite its low tensile performance of about 1500% [127]. Furthermore, ionic hydrogels that are physically cross-linked demonstrate exceptional qualities, including a super-stretch ability, easy plasticity, and self-healing capabilities, which enable them to adapt to the dynamic surface of the skin and promptly recover when damaged [128], which implies the potential of the next generation of intelligent touch panels.

First, physical cross-linking between poly(*N*, *N*′-dimethylacrylamide) chains and silica nanoparticles in a lithium chloride solution creates an ultra-stretchable, self-healing composite hydrogel. In its prepared state, the hydrogel exhibited mechanical and electrical self-healing characteristics and a fracture elongation of up to >3800%. The composite hydrogels were then used to create an ionic surface capacitive touch panel, which demonstrated continuous self-healing-sensing capabilities. Writing words and playing computer games served as demonstrations for the skin touch panel’s construction. Additionally, these hydrogels will encourage the creation of intelligent skin-associated devices for future generations, particularly for use cases where the human body must endure severe acceleration, stretching, and impact.

**Figure 11:**
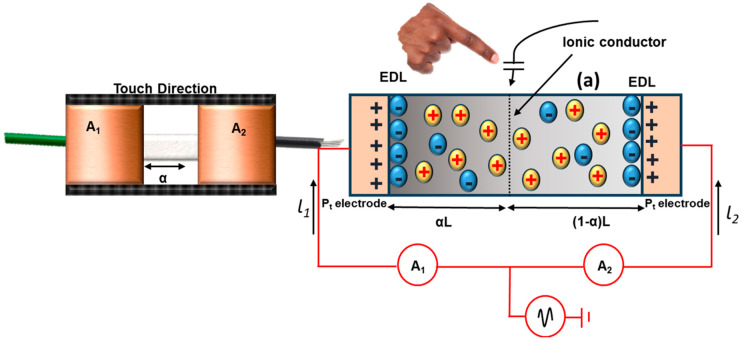
Illustration of central touch analysis to find the connections between the current versus touch location of 1D touch strip.

The platinum (Pt) electrode associated with both the terminal of the gel strip and the alternating current was applied to all the terminals of the gel strip, as stated in Figure 11. The touch panel functions as the positive source, and when a human touches it, it connects to the ground or the negative. Once the circuit is complete, the touch panels generate a potential difference that induces a current through the finger. Additionally, the strip functions as a parallel circuit with two resistive components, and in response to hydrogel interference, it forms a coupling capacitance that permits the current to flow from the electrodes to the touch position via the gel strip. The distance between the electrode and the touch site also determines the current’s amplitudes. The C-touch capacitor splits the gels into two divisions by touching the finger to the touch panel. In an electric double layer (Cedl), a capacitor connects the gels and ammeters in series, with the two components connected in parallel and in series to the capacitor. Due to the high AC frequency and the capacitor’s broad electric double layer, we neglected the electric double layer in the parallel AC circuit [129]. Figure 11 illustrates the parallel touch experiment conducted to confirm the connection between the touching current and touch location. The touching current was measured by touching the finger for approximately 5 s, at intervals of 10 mm, in an onward direction towards the touch strip. The parasitic capacitance between the strip and the environment develops the baseline current, which is considered the leakage current in the order of microampere. Table 3 presents the hydrogel’s touch panels, which are widely used in e-skin and wearable electronics where the performance of hydrogels in touch panels is satisfying.

### 3.4. Hydrogels in Solar Cells

**Figure 12:**
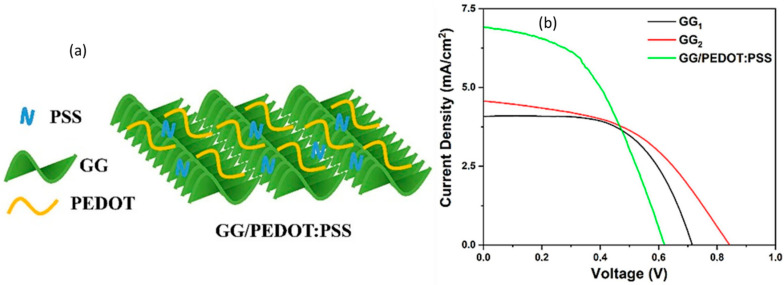
(**a**) GG/PEDOT:PSS model and (**b**) comparison curves of I–V for the different gel electrolytes in DSSCs (reused with permission [140]).

To promote efficiency, hydrogels of suitable components are being tried for use in solar cells. A dye-sensitized solar cell’s performance depends on the constituent components of the device. Usually, organic solvent-based electrolytes are highly efficient, even though they have some limitations, including flammability, volatility, and leakage. Here, the solvent water faces lower voltage problems (0.4–0.6 volts). To mitigate those problems, hydrogels are employed as water-based quasi-solid electrolytes that have both liquid and solid properties [136,137]. The liquid electrolytes corrode the conductive materials and electrodes used in solar cells, resulting in leakage and evaporation of the solvent [138]. Zarate et al. successfully implemented zinc–galactomannan hydrogel electrolytes in dye-sensitized solar cells whose design was green, and showed an open-circuit voltage of 750 mV [139]. Unlu et al. prepared gellan gum/poly(3,4-ethylenedioxythiophene)/polystyrene sulfonate (GG/PEDOT:PSS) gel and immersed it in an I^−^/I_3_^−^ solution to use as an electrolyte in quasi-solid dye-sensitive solar cells (DSSCs). On the basis of thickness, different samples of gels—GG_1_ (2 mm), GG_2_ (1 mm), and GG/PEDOT:PSS—were prepared for an efficiency comparison. The conjugated polymeric 3D gels and free ions in the DSSCs maintain the electron transfer for electricity. Figure 12 shows the GG/PEDOT:PSS gel network and I–V (current density versus voltage) curves for the GG_1_, GG2, and GG/PEDOT:PSS gel electrolytes, where the last one exhibits the best short-current density (Jsc) over the others, which are 4.08, 4.57, and 6.92 mA/cm^2^, respectively [140]. Table 4 lists the recent solar cells that use hydrogels as their important component, as well as improved efficiency up to 14%.

### 3.5. Hydrogels in Flexible Energy Storage and Batteries

**Figure 13:**
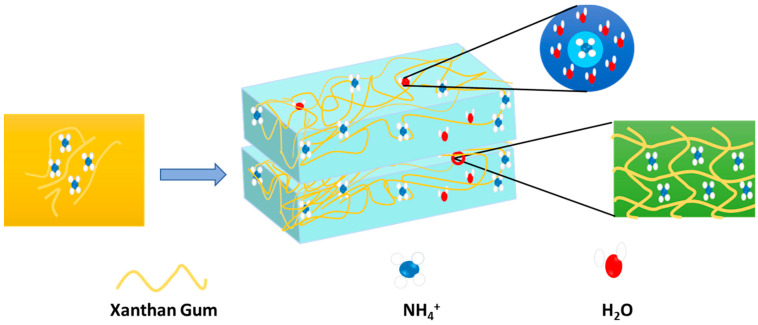
Hydrogel network as electrolyte for battery flexible battery system.

Hydrogel has been investigated and evaluated as a potential cathode and anode for supercapacitors for high-power density energy storage. The charge and discharge cycles are made possible by the hydrogel’s interconnected porosity structure, which provides an improved and effective surface area for ion storage [150]. In this case, hydrogels can be chemically altered or doped with conductive material to improve their overall performance as supercapacitor electrodes, as well as their effectiveness and efficiency. Since conductive hydrogels can be employed as electrodes or electrolytes in these devices, which offer high capacitance, quick charging and discharging rates, and long-term stability, hydrogels have also been investigated for application in supercapacitors and batteries [151]. Because of their unique properties and structure, hydrogels are revolutionizing energy storage technology and changing the game in the field of solid-state supercapacitors [152]. Enhancing the mechanical flexibility and durability of solid-state supercapacitors is one of the most popular uses of hydrogel. Hydrogel-based supercapacitors are perfect for flexible and wearable electronics because they come in a variety of shapes and sizes, unlike traditional supercapacitors that rely on rigid materials [153]. Wearable bioelectronics are being developed to accommodate biological tissue and increase the immune system. Due to the high Young’s modulus of traditional batteries, they are not suitable for adjusting to the soft tissue of the living body. In contrast, tissue-like batteries made of soft hydrogels match the skin, heart, and other organs of the living human body. Nowadays, the stability and biocompatibility of hydrogels make them suitable for wearable and implantable devices for the body [154]. The use of metals in metal-oriented battery preparation reduces natural resources and increases the detrimental risk to the soil environment. The replacement of metals by organic compounds and hydrogels may bring a revolution to the energy sector [155]. The compressibility of hydrogel electrolytes is highly demanding for flexible batteries. A protein isolate nanoparticle–acrylamide hydrogel electrolyte showed extraordinary reversible compressibility when used in a Zn–MnO_2_ battery; consequently, the battery displayed a 299.3 mA h g^−1^ specific capacity with a capacity retention rate of 78.2% after 500 charge and discharge cycles and almost 100% coulombic efficiency at 0.4 C. The outcome is nearly identical to that of a regular Zn–MnO_2_ battery [156,157]. Hydrogel-fabricated ammonium-ion batteries can be an alternative to lithium-ion batteries, where NH_4_^+^ acts as a charge carrier. Recently, Paudel et al. made a biodegradable hydrogel-based flexible ammonium-ion battery without incorporating metal. Figure 13 presents the details of hydrogel electrolytes in a flexible battery.

In this battery, a hydrogel electrolyte prepared from ammonium sulphate and xanthan gum was sandwiched in between the polyaniline anode and the polypyrrole cathode [158]. The capacity of this battery is 44.321 mA h g^−1^, with 74.56% retention for 100 cycles at 0.1 A g^−1^. It also exhibits excellent bending and twisting mechanical deformation performance. The hydroxyl and carboxyl groups of xanthan gum interact with ammonium ions to facilitate diffusion and intercalation. The embedded ammonium ions play a role in the conductance of the gel electrolytes. Here, the partial negative charge of oxygen in water attracts the hydrogen of ammonium ions. In parallel, the partial positive charge of hydrogen in water attracts nitrogen in the ammonium ions. Thus, the hydration shell is established, which improves the mobility of charges. The entrapment of the hydrogel electrolyte in flexible batteries reflects the electrochemical performance through a glowing LED bulb. In Table 5, the name of hydrogels is mentioned, along with their constituent raw materials and applicable types of batteries. The performance of the hydrogel and its role in the battery are progressive. 

### 3.6. Hydrogels in Soft Robotics

**Figure 14:**
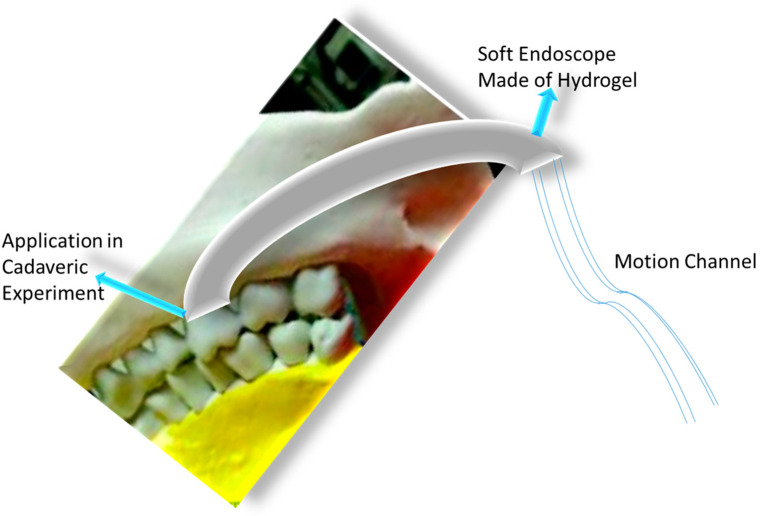
Agar/polyacrylamide DN hydrogels as soft endoscope.

Usually, soft robots use silicone as a constituent component. Nowadays, for their excellent mechanical strength, double- or triple-network structure, ultra-low viscosity, and transparency, hydrogels are considered an alternative to silicone and are used in soft robotics. Despite having a greater resistivity than other soft electrical materials, hydrogels have electrical resistance increases with strain that are orders of magnitude less because configuration changes in polymer networks rarely impact the movement of mobile ions. Hydrogels can therefore be employed as electrodes in applications requiring a high degree of stretchability, such as soft robotics. Yet, there are some limitations to using hydrogels for soft robot construction, which include difficulties in reproducibility and recovering softness due to swelling and degradation. Usually, agar, polyethylene glycol, gelatin, acrylamide, and similar materials make hydrogels suitable for soft robots. Banerjee et al. prepared a agar/polyacrylamide-based double network (DN) hydrogel for soft robots, where agarose forms the first network and, upon photo-initiation, the acrylamide forms the second network to give the final product for pneumatic robotic application. The hydrogels show the load bearing and elasticity required for a soft nasal endoscopic robot, as exhibited in Figure 14. This DN hydrogel bears around 1800% strain and about 300 kPa tensile stress, which are much better than silicone, indicating its suitability for use in soft robots. The DN gels (3 mm diameter with 1 mm cavity and wounded with threats) connected with flexible tubes are usable as actuators in soft nasal endoscopic soft robots. Figure 14 explains the successful implementation of a soft endoscopic robot in a human cadaver skull, which conveniently demonstrates agar/PAM as an alternative to other rigid materials [169]. By choosing better constituent compounds, the limitations of present soft robots can be mitigated to provide next-generation instruments with the best performance. Different types of hydrogels used in soft robotics are listed in Table 6, which clearly presents the applicability of hydrogels as a part of a robot or as a whole robot.

### 3.7. Gels in Thermal Insulators

The interaction of solar light with gels and other materials leads to the conversion of photon energy into thermal energy through a process known as light-to-heat conversion. Materials with high thermal conductivity facilitate the transfer of this heat to areas with lower thermal zones, while materials with lower thermal conductivity act as insulators. Due to this principle, gels, mainly aerogels with low thermal conductivity, are now commonly used as thermal insulators in various electronic devices. Aerogels are a type of open-cell, mesoporous foam that possess exceptional physical and chemical properties, such as ultra-high porosity (up to 99.9%), significantly large internal void spaces, low density, and excellent thermal insulating capabilities. As a porous amorphous solid, aerogels exhibit promising advantages in reducing solid heat conduction and limiting thermal convection within their porous structure. They have demonstrated 0.02 W m^−1^ K^−1^ thermal conductivity at an ambient temperature of 25 °C, which is much lower than other conventional commercial insulators, like mineral wool and polymer foam (thermal conductivity around 0.03–0.04 W m^−1^ K^−1^) [179,180]. Due to their unique properties, aerogels are being used as photothermal insulators in photothermal materials for solar steam generation. Dong et al. reported photothermal materials made from SiO_2_ aerogel and CNT that can be used as an effective solar steam generator, where SiO_2_ aerogel plays a role as a thermal insulator for heat localization [181]. The fireproof and heatproof grade of silica aerogel (SA) can be achieved as a construction material of class A1, which means it will not produce smoke, droplets, or hazardous gases when exposed to a flame. Silica aerogel is now being used in building paints for thermal insulation, which affects the reduction in temperature with increasing volumes of silica aerogel. Altay et al. prepared two materials painted with and without the presence of SA and tested them for heat reduction. After heating, they found that with SA, the heat reduction properties of the paint had improved (up to 15 °C). In the manufacturing of firefighting gear and comfortable clothing with high thermal resistivity, aerogels show good performance [182]. Guo et al. synthesized ceramic-based hypocrystalline zircon nanofibrous aerogels (ZAG) with a zig-zag architecture, which possessed a super thermomechanically stable and high-temperature thermal insulation [183].

**Figure 15:**
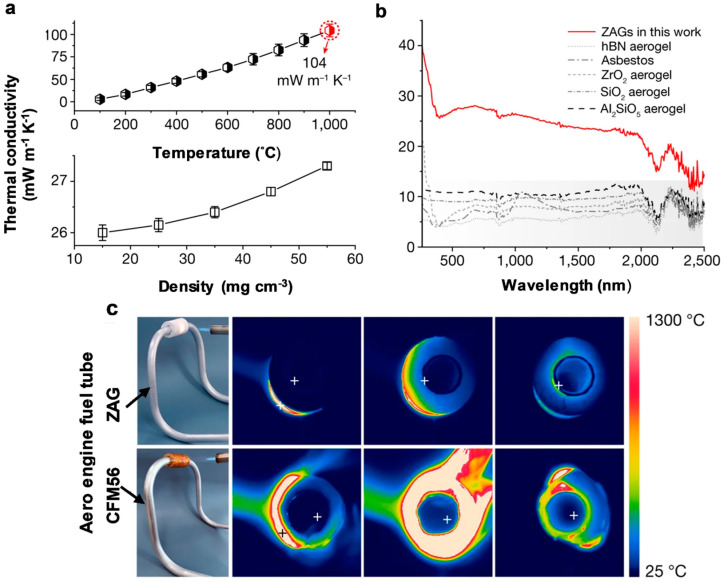
Thermal stability and insulation characteristics. (**a**,**b**) Relationship of thermal conductivity with density, temperature, and wavelength; (**c**) comparison of thermal insulation between ZAG and commercial barrier for an aero-engine (CFM56) [183].

Figure 15a,b illustrates variations in thermal conductivity concerning changes in the temperature, density, and wavelength of nanofibrous zirconium aerogel materials (ZAG) in their prepared state. In accordance with general thermodynamic principles, thermal conductivity generally increases with rising temperature. However, as shown in Figure 15a, the increase in thermal conductivity of ZAG is only 104 mW m^−1^ K^−1^ and 28 mW m^−1^ K^−1^ at temperatures around 1000 °C and densities around 55 mg cm^−3^, respectively. In addition, the thermal conductivity of ZAG has been compared with different materials for variations in wavelength. It also shows a correlation with increasing radiation energy. Additionally, the material was tested as thermal insulation for an aero-engine tube, compared to the commercially used CFM56 tube. It is evident from Figure 15c of the data that the temperature increase of ZAG is lower than that of the CFM56 tube. The unique properties of ZAG make it suitable for use in spacecraft, deep-earth detectors, furnaces, and space and fire suits. Recent studies have also explored the use of gels as thermal insulators in electronic devices. Small electronic devices and metal oxide (MOX) gas sensors need materials with low thermal conductivity, such as electrical insulators. Ordinary low-thermal-conductivity materials require high-temperature treatment. Here, gels can be used in thermal insulation systems. Fagnard et al. developed a xerogel–epoxy composite for use in electronics, where they found thermal conductivity as low as 64.2% compared to pure epoxy, that is, 107.9 mW m^−1^ K^−1^ [184].

## 4. Conclusions

The next generation of devices and instruments demands flexible, self-healing, and wearable properties to make them user-friendly. On the basis of unique characteristics, hydrogels are chosen for constructing specific devices like sensors, actuators, soft robots, solar cells, and flexible energy equipment. This review article discussed the state of the art of different devices using hydrogels as a significant part, the advantages of using hydrogels rather than other materials, and the scope of improvement. The motion of human organs can be detected using gel-based sensors. Actuators convert input energy into mechanical energy that is usable in various machines. Flexible energy storage and touch panels are the most significant parts of devices in this smart era that use hydrogels as essential constituent elements. The replacement of metal-ion batteries with hydrogels can be a revolutionary sector that is in progress. Due to their double or triple network structure, ultra-low viscosity, and transparency, hydrogels are replacing the silicon constituent components of soft robots. Solar cells are a highly demanding research area in energy. Hydrogels play an important role in the construction of efficient solar cells. The working mechanism of hydrogels in various devices has been illustrated with relevant figures and graphs. The eligible raw materials, preparation methods, and properties of hydrogels were presented through figures. Recent research on hydrogel-based devices has been listed in tables, along with their performance. Finally, it can be stated that hydrogels are emerging materials for the better future of different devices. 

## Figures and Tables

**Table 1:** Different hydrogels in sensors.

S.N.	Name of Hydrogel	Characteristics	Types of Sensors and Applications	Performance	References
01	MXene poly(acrylic acid) (PAA)–amorphous calcium carbonate composite hydrogel	Excellent stretch ability, recyclability, favorable shape adaptability, adhesiveness	Pressure sensor. For e-skin.	1. Conductivity 0.8 S m^−1^ 2. Tensile strain >900%	[81]
02	Cationic cellulose nanofibers (CCNFs)–liquid metals (LM)–poly(acrylic acid) hydrogel	Good conductivity, mechanical property, self-adhesiveness, quick self-healing	Strain sensor. For monitoring human body movement.	1. Conductivity 1.54 S m^−1^ 2. Tensile strain >1500%	[82]
03	Poly(Vinyl alcohol) (PVA)–glutaraldehyde/poly(acrylic acid-*co*-Acrylamide) double network hydrogel	High adhesiveness, sensitivity, temp. tolerance	Stain and pressure sensor. For monitoring human motion and physiological activities.	1. Conductivity 0.83 S m^−1^ 2. Tensile strain 1700%	[83]
04	Chitosan-poly(Acrylamide-*co*-acrylic acid) double network hydrogel	Good mechanical properties, conductivity, durability, strong freezing tolerance	Strain and pressure sensor. For biomimetic, skin health monitoring, and soft robots.	1. Conductivity 0.32 S m^−1^ 2. Tensile Strain ~450%	[84]
05	Gelatin(G) carboxylated carbon(C) polypyrrole (PPy)–gold (AU) hydrogel	Good mechanical properties, electroconductivity, biocompatibility	Pressure sensor. For e-skin.	1. Conductivity 2.33 S m^−1^ 2. Tensile strain 253%	[85]
06	Poly(Vinyl alcohol) (PVA)/poly(Acrylamide-*co*-acrylic acid)-Fe^3+^ double network hydrogel	Maximum open-circuit voltage, short-circuit current, short-circuit transferred charge	Strain sensor. For wearable devices, human health monitoring, and energy harvesting.	1. Toughness 6.5 MJ m^−3^ 2. Elasticity modulus 0.4 MPa	[86]
07	Poly(Vinyl alcohol) (PVA)–cellulose nanofibers (CNF) hydrogel	Highly stretchable, strong, tough, transparent, and ionic conductive	Multi-functional strain and pressure sensor. For detecting human body movement.	1. Toughness 5.25 MJ m^−3^ 2. Elasticity modulus < 1.1 MPa	[87]
08	Double network hydrogel	Good biocompatibility, stretch ability, self-healing property	Strain sensor. For human and organ motion.	Self-healing efficiency 95.3%	[88]
09	Poly(Vinyl alcohol) (PVA)–borax(B)–sodium alginate (SA)–tannic acid (TA) hydrogel	pH sugar responsiveness, high stretch ability, high healing ability	Strain sensor. For detecting human motion.	Self-healing efficiency 93.56% in 10 min	[89]
10	Poly(Vinyl alcohol) (PVA)–MXene poly(3,4-ethylenedioxythiophene):poly(4-styrene sulfonate) hydrogel	Good biocompatibility	Multi-functional strain and position sensor. For human motion detection, detecting speed sensor, and hand-writing recognition.	Self-healing efficiency 95.47% in 30 min	[90]
11	Dried smart poly(*N*-isopropylacrylamide) hydrogel	Stable, porous structure, large surface area	Gas sensor	Exhibits a high affinity for various organic gases	[91]

**Table 2:** Different hydrogels in actuators.

S.N.	Name of Hydrogel	Characteristics	Types of Actuators and Applications	Performance	References
01	Poly(*N*-isopropylacrylamide)–clay nanocomposite (NS) hydrogel	Rapid, reversable, repeatable thermos-responsive bending	Thermo-responsive. Actuator for encapsulation, capture, and transportation.	Bending angle 180°/120 s	[105]
02	Poly(*N*-isopropylacrylamide)/poly(2-(dimethylamino)ethyl methacrylate)–acrylamide (AM) hydrogel	Temperature/salt- and temperature/pH-responsive	Bilayer and multiple stimuli-responsive. Actuator for soft robotics, biometric devices, and environmental sensors.	Bending angle 180°/60 s	[106]
03	Alginate-Poly(*N*-isopropylacrylamide)/Aluminum-alginate-Poly(*N*-isopropylacrylamide) hydrogel	High mechanical properties	Bilayer and thermo- responsive. Actuator for soft robotics.	Bending angle 140°/50 s	[107]
04	Poly(*N*-isopropylacrylamide)-poly(Vinyl alcohol)/poly(2-(dimethylamino)ethyl methacrylate)–poly(sodium-p-styrenesulfonate) hydrogel	Rapid, reversable, repeatable bending motion	Bilayer and intelligent responsive actuator. Soft actuator.	Bending angle 180°/60 s	[108]
05	Poly(*N*-isopropylacrylamide)/poly(Acrylic acid-*co*-acrylamide) hydrogel	Self-water circulation, reversible actuation	Bilayer and thermo-responsive. Actuator for soft material.	Bending angle 180°/60 s	[101]
06	Poly(*N*-isopropylacrylamide)/poly(3-(1-(4-vinylbenzyl)-1*H*-imidazol-3-ium-3-yl) propane-1-sulfonate) hydrogel	Fast, reversible, bidirectional bending behavior	Bilayer and thermo-responsive. Actuator for soft and intelligent material.	Bending angle 400 °	[109]
07	Poly(Acrylic acid)/carboxylic reduced graphene oxide/Fe hydrogel	High stretch ability, self-healing ability, rapid bending actuation	Electro-responsive. Actuator for soft robots.	Bending angle > 150°/180 s	[110]
08	Carbon nanotube/poly((2-acrylamido-2-methyl-1-propane-sulfonic acid)-*co*-acrylic acid) hydrogel	Good electric conductivity	Electro-responsive. Strain actuator.	Bending angle 90°/120 s	[111]
09	Poly(*N*-isopropylacrylamide)-*co*-hydroxyethyl acrylate–laponite hydrogel	Fast response to hot water, rapid recovery in air	Thermo-responsive. Actuator for soft robots, micromanipulation, microfluidics, and artificial muscles.	Bending angle 100°/40 s	[112]
10	Poly(2-acrylamido-2-methyl-1-propane–sulfonic acid)/polypyrrole hydrogel	High elasticity, high bending rate	Electro-responsive. Actuator for electric field.	Time 40 s	[113]

**Table 3:** Hydrogels for touch panels.

S.N.	Name of the Hydrogel	Characteristics	Type of Touch Panel	Efficiency	Reference
01	Poly(*N*-isopropylacrylamide)–poly(vinyl alcohol)/sodium acrylate hydrogel	Stretchability, self-healing ability	Optical touch panel. For e-skin wearable electronics and smart windows.	Optical transparency 91%, stretch ability (150% to 600%)	[126]
02	Polyacrylamide hydrogel	Stretchability, biocompatibility	Capacitive touch panel. For e-skin.	Optical transparency 98%, stretch ability (>1000%)	[129]
03	Polyacrylic acid-polycation (poly(methyl chloride quaternized N < N-dimethyl-amino ethyl acrylate)) hydrogel	Self-healing ability, self-power voltage ability	Optical touch panel. For soft robotics and artificial intelligence.	Optical transparency 90%, stretch ability (>10,000%)	[130]
04	k-carrageenan/poly(*N*-acrloyl glycinamide (NAGA)–co-vinyl imidazole (VI)) hydrogel	Thermoplasticity, injectability, tough, fast self-recovery, thermal degradation resistance, durability, controllable adhesion	Resistive touch panel. For sensing devices.	Stretch ability (1045%)	[131]
05	Titanium dioxide/poly(*N*, *N*′-dimethylacrylamide) hydrogel	High stretch ability, soft, low parasite capacitance, high resolution, fast response	Capacitive touch panel. For e-skin.	Transparent stretch ability (1100%)	[115]
06	Silicon dioxide/lithium cation/poly(*N*, *N*′-dimethylacrylamide) hydrogel	Ultra-stretch ability, self-healing	Capacitive touch panel. For e-skin.	Stretch ability (>3800%)	[53]
07	Glycerin and hydroxyethyl cellulose elastomer and poly-acrylamide/carrageen hydrogel	Self-healing without degradation, good mechanical property, durability	Optical touch panel. For touch screen sensors.	Optical transparency 93%, stretch ability (310% for GHEC elastomer, 906% for PAM Carrageen)	[132]
08	Gelatin–poly acrylic acid (PAA)-based organic hydrogel	Excellent self-adhesion, self-healing, anti-freezing, anti-drying	Optical touch panel. For health care and human–machine interface.	Optical transparency 87%, stretch ability (1700% at 60 °C and 1200% at −20 °C)	[133]
09	Polyvinyl chloride ion gel	Good stretch ability, high transparency	Optical flexible touch panel. For smart electronic devices.	Optical transparency 90%, stretch ability (250%)	[134]
10	Poly(3,4-ethylenedioxythiophene):poly(styrene sulfonate) PEDOT:PSS ionogel	Mechanical and electrical conductivity and good transparency	Optical touch panel. For electronic and optoelectronic devices.	Optical transparency 87%, stretch ability (200%)	[135]

**Table 4:** Hydrogels in different solar cells.

S.N.	Type of Gel	Characteristics	Type of Solar Cell	Photoelectric Conversion Efficiency	Reference
01	Poly(acrylic acid-*co*-acrylamide)/polyaniline hydrogel	Enhanced diffusion and reduction in iodine ions	Dye-sensitized solar cells	2.0%	[141]
02	Graphene oxide/gelatin hydrogel	High open-circuit voltage	Quasi-solid-state dye-sensitized solar cells	4.02%	[142]
03	Polyacrylamide/bis-acrylamide hydrogel	High absorbent ability	Quasi-solid-state quantum-dot-sensitized solar cell	4.3%	[143]
04	Polyvinyl alcohol/multiwall carbon nanotube/polyaniline hydrogel	Good ionic conductivity charge transportation	Dye-sensitized solar cells	2.18%	[144]
05	Polysaccharide dextran hydrogel	Good light intensity	Quasi-solid-state quantum dot-sensitized solar cells	4.58%	[18]
06	Polysulfide hydrogel	Long-term stability	Quasi-solid-state quantum-dot-sensitized solar cells	2.40%	[145]
07	Graphene hydrogel	Good stability	Quantum-dot-sensitized solar cells	10.71%	[146]
08	Carbon nanotube/graphene hydrogel/copper sulfide	Good conductivity, catalytic activity	Quantum-dot-sensitized solar cells	14.02%	[147]
09	Chlorophyll–a/polyacrylamide hydrogel	High pigment absorption	Biomimetic solar cells	Power conversion efficiency is 0.59%.	[148]
10	Poly(m-amino benzodioxol)–iron (ii, iii) oxide nanorods hydrogel	High thermal stability and photovoltaic properties	Hybrid solar cell	6.08%	[149]

**Table 5:** Hydrogels for batteries.

S.N.	Name of Hydrogel	Characteristics	Type of Battery	Performance	References
01	Carboxymethyl cellulose (CMC) hydrogel	Low cost, high cycling ability	Zn-ion batteries	1. Conductivity 34.5 mS cm^−1^ 2. Mechanical strength 1.33 MPa	[159]
02	Chitosan–Zn membrane electrolyte	High conductivity, non-flammability	Zn-metal batteries	1. Conductivity 71.8 mS cm^−1^ 2. Mechanical strength 7.4 MPa	[160]
03	Kappa (k)-carrageenan–chitosan hydrogel	High cycling stability, mechanical strength	Zn-metal batteries	1. Conductivity 5.3 mS cm^−1^ 2. Mechanical strength 14.2 MPa	[161]
04	Natural chitosan–glass fiber hydrogel	High conductivity	Zn-ion batteries	1. Conductivity 83.4 mS cm^−1^ 2. Mechanical strength 2.40 MPa	[162]
05	Gelatin-based hydrogel electrolyte	High conductivity, easy fabrication	Zn-metal batteries	1. Conductivity 37.2 mS cm^−1^ 2. Mechanical strength −100 MPa	[163]
06	Xanthan gum hydrogel	High conductivity, easy fabrication	Zn-ion batteries	1. Conductivity 14.6 mS cm^−1^	[164]
07	Cellulose nanofiber–polyacrylamide (PAM) hydrogel electrolyte	High cycling stability, wide temperature stable window	Zn-ion batteries	1. Conductivity 6.8 mS cm^−1^ 2. Mechanical strength 192 MPa	[165]
08	Polyacrylamide (PAM)–cotton cellulose nanofiber–carboxymethyl cellulose (CMC) hydrogel	Very high conductivity, high stretchability	Zn-ion batteries	1. Conductivity 2.492 S m^−1^ 2. Mechanical strength 60 MPa	[166]
09	Xanthan gum g-cellulose nanofiber/cotton cellulose nanofiber hydrogel	High mechanical strength and good adhesion	Zn-ion batteries	Ionic conductivity 28.8 mS cm^−1^, tensile strength of 84 kPa	[167]
10	Poly(2-acrylamido-2-methyl propane sulfonic acid potassium salt)/methyl cellulose hydrogel	Rapid self-recovery, good toughness, and antifatigue properties	Zn–air batteries	Ionic conductivity 105 mS cm^−1^, compressive strength of 170 kPa	[168]

**Table 6:** Hydrogels in different soft robots.

S.N.	Name of Hydrogel	Characteristics	Type of Robotics	Performance	References
01	Poly(*N*-isopropylacrylamide)/graphene oxide hydrogel	Fast bending actuation	Soft robotics	1. Response time 16–24 s 2. Tensile strength 83 kPa 3. Bending motion	[170]
02	Poly(*N*-isopropylacrylamide)/poly(acrylic acid-*co*-acrylamide) hydrogel	Reversible actuation	Soft robotics	1. Response time ~60 s 2. Bending motion	[101]
03	Chitosan/carboxymethyl cellulose hydrogel	Rapid, reversible, bidirectional deformation	Soft robotics	1. Response time 240 s 2. Tensile strength 62 kPa 3. Coiling motion	[171]
04	Poly(*N*-isopropylacrylamide)/graphene oxide hydrogel	Excellent comprehensive actuation	Soft robotics	1. Response time 40 s 2. Bending motion	[172]
05	Poly(*N*-isopropylacrylamide-*co*-Ru(bpy)_3_^2+^)-ruthenium(ii) tris(2,2-bipyridine)-*co*-hydrophilic 2-acrylamide-2 methylpropane sulfonic acid hydrogel	Repeated bending and stretching motion	Biometric robotics	1. Tensile strength 410 kPa	[173]
06	Poly(*N*-isopropylacrylamide)/2-acrylamido-2-methylpropane sulphonic acid hydrogel	Different shrinkage and elastic moduli on applied external stimuli	Programmable soft robotics	1. Response time 2 h 2. Twisting motion	[174]
07	Polyethylene glycol diacrylate/gelatin methacrylate-*co*-Polyethylene glycol dimethacrylate hydrogel	Shape-change ability	Soft robotics	1. Tensile strength 20.3/1.8 kPa 2. Folding and twisting motion	[175]
08	Poly(*N*-isopropylacrylamide)/polyethylene glycol diacrylate hydrogel	Untethered, self-folding	Micro transport robotics	1. Response time 60–120 s 2. Bending and rotating motion	[176]
09	Graphene oxide–polydimethylsiloxane/polydimethylsiloxane hydrogel	Excellent stability	Soft robotics	1. Response time 1–5 s 2. Swimming motion	[177]
10	Graphene oxide/graphene oxide–polydopamine hydrogel	Light sensitivity	Soft robotics	1. Response time 1.5 s 2. Crawl motion	[178]

## Data Availability

No new data were created or analyzed in this study.
